# Pediatrics in the era of precision medicine and precision epidemiology

**DOI:** 10.1002/pdi3.28

**Published:** 2023-09-06

**Authors:** Janne Estill

**Affiliations:** ^1^ Institute of Global Health University of Geneva Geneva Switzerland; ^2^ School of Basic Medical Sciences Lanzhou University Lanzhou China

**Keywords:** epidemiology, omics, precision epidemiology, precision medicine, public health

The advances in different fields of science, in particular the “omics,” have enabled a revolution in the approach to medical care. Until recently, healthcare in the global context could be roughly divided into two types. Individuals living in wealthy settings or having adequate insurance coverage could expect highly individualized care, consisting of a thorough procedure through diagnosis to selecting the optimal therapy and follow‐up. On the other hand, the “public health approach”—providing simplified and uniform diagnostic and treatment protocols to be implemented on a massive scale at a short time window— has led to remarkable progress, e.g., in the management of the HIV epidemic, making HIV essentially from a certain death sentence into a manageable chronic condition in the most heavily affected regions of sub‐Saharan Africa. The recent COVID‐19 pandemic showed that the “public health approach”—for example, the introduction of society‐wide nonpharmaceutical interventions and the mass vaccination of the entire population—may also be unavoidable in settings with good resources and highly developed healthcare systems in unexpected emergency situations.

The concepts of “personalized medicine” and “precision medicine” became increasingly topical in the years preceding the pandemic.^1^ The idea behind these concepts is that most health conditions result from a complex network of cofactors that are at least to some extent quantifiable. The exponentially increasing amount of available data—ranging from socioeconomic to various genomic indicators—and the rapid development of new data analysis tools provide revolutionary opportunities for selecting the optimal management and treatment for each individual. The development of precision medicine has also given rise to another related concept, precision epidemiology.^2^ The new tools for precision epidemiology are not only available for guiding clinical practice and the care of individual patients but also for public health on the larger scale—for example, to plan prevention and screening programs and control the spread of infectious diseases. For example, in the early stage of the COVID‐19 pandemic, Koks and colleagues proposed a seven‐point action plan for applying the precision epidemiology approach in the management of the pandemic.^3^ Although precision epidemiology is often understood as involving huge datasets with a large number of variables, the same approach can also be used to identify much simpler predictive pathways that do not necessarily require the conduction of time‐ and resource‐consuming laboratory analyses—for example, by identifying combinations of easily observable characteristics that can be used to form risk profiles.

## 1 WHERE DOES PEDIATRICS STAND IN THE UTILIZATION OF PRECISION TOOLS FOR EPIDEMIOLOGICAL AND CLINICAL RESEARCH AND PRACTICE?

The value of precision medicine is also increasingly acknowledged in the field of pediatrics. A search in MEDLINE with the term “pediatrics” together with “precision medicine” revealed almost 7000 records. In contrast, only one publication that clearly applied precision epidemiology into research could be identified. The situation shows that the value of precision methods is acknowledged; however, in particular, in the field of epidemiology, there is yet no standardized framework that could help to maximize the benefits of this approach.

The current issue of *Pediatric Discovery* also contains research that demonstrates new opportunities that could lead to major benefits if integrated into a well‐structured ecosystem of precision epidemiology. Xia and Peng present an overview of perinatal transmission of SARS‐CoV‐2, which is a complex issue linked with the health of both the mother and the infant, including three essentially different routes of transmission, and a variety of factors associated with both the mother's and infant's risk of developing severe symptoms.^4^ The prevention is therefore extremely challenging. In the post‐pandemic era where the highly transmissible SARS‐CoV‐2 variants continue in circulation, complete protection of all pregnant women from infection is an implausible task. But if the factors that have the highest impact on the risk and consequences of a possible SARS‐CoV‐2 infection can be identified, protection strategies can be tailored for the different individual situations—aiming at finding a balance between providing the maximal protection to the mother and child from COVID‐19‐related sequelae and the possibility of the other to live a life free of unnecessary restrictions in the very sensitive life situation. As another example, Zhong and colleagues provide a thorough review of possible treatment opportunities for glycogen storage disease type I—a rare disorder that has previously been managed primarily by nutritional interventions.^5^ While rare diseases have been to some extent neglected in the past due to resource limitations and lack of high‐quality evidence, this new knowledge opens new opportunities. Early identification of the risk factors together with a combination of both traditional and innovative treatment options can provide new opportunities to treat the rarer conditions effectively and efficiently.

## 2 TOWARDS AN INTEGRATED FRAMEWORK OF PRECISION EPIDEMIOLOGY IN PEDIATRICS

Pediatrics is an extremely complex field that does not only overlap with most branches of medicine but also with other fields of science, such as social and behavioral sciences, pedagogics, ethics, environmental research, and law. Because of this complexity, new multidisciplinary methods and approaches are particularly needed in pediatrics. We should strive to bring together the knowledge from basic sciences and “omics,” data science, clinical practice, and epidemiological theories to build a framework that can guide both health policy makers and clinical practitioners in making decisions that are optimal for the individual in an efficient manner: for example, to screen children or newborns with severe or rare conditions and provide an optimal therapy; manage infectious disease epidemics so that everyone can be optimally protected with minimal restrictions in their daily life; understand the influence of the current environmental challenges on child health; or identify children or adolescents particularly vulnerable to certain health or mental conditions. The theory is already there—what is now urgently needed is the integration of this theory from different fields into a precision epidemiology ecosystem, which in turn will facilitate the successful implementation of this new knowledge into clinical practice and health policy.

## AUTHOR CONTRIBUTIONS

Conceptualization, literature search and writing the manuscript: Janne Estill.

## CONFLICT OF INTEREST STATEMENT

The author serves as the Deputy Editor‐in‐Chief of *Pediatric Discovery*. To minimize bias, he was excluded from all editorial decision‐making related to the acceptance of this article for publication.

## ETHICS STATEMENT

Not applicable.

## Data Availability

Data sharing is not applicable to this article as no datasets were generated or analyzed during the current study.
